# High-temperature cultivation of recombinant *Pichia pastoris* increases endoplasmic reticulum stress and decreases production of human interleukin-10

**DOI:** 10.1186/s12934-014-0163-7

**Published:** 2014-11-26

**Authors:** Yongjun Zhong, Lu Yang, Yugang Guo, Fang Fang, Dong Wang, Rui Li, Ming Jiang, Wenyao Kang, Jiajia Ma, Jie Sun, Weihua Xiao

**Affiliations:** The CAS Key Laboratory of Innate Immunity and Chronic Disease, Innovation Center for Cell Biology, School of Life Sciences, University of Science and Technology of China, Hefei, China; Hefei National Laboratory for Physical Sciences at Microscale, Engineering Technology Research Center of Biotechnology Drugs, Anhui Province, University of Science and Technology of China, Hefei, China

**Keywords:** High-temperature cultivation, Misfolded protein, ER stress, Unfolded protein response, ER-phagy, *Pichia pastoris*

## Abstract

**Background:**

The yeast *Pichia pastoris* (*P. pastoris*) has become a popular ‘cell factory’ for producing heterologous proteins, but production widely varies among proteins. Cultivation temperature is frequently reported to significantly affect protein production; however, the underlying mechanisms of this effect remain unclear.

**Results:**

A *P. pastoris* strain expressing recombinant human interleukin-10 (rhIL-10) under the control of the *AOX1* promoter was used as the model in this study. This system shows high-yield rhIL-10 production with prolonged methanol-induction times when cultured at 20°C but low-yield rhIL-10 production and higher cell death rates when cultured at 30°C. Further investigation showed that G3-pro-rhIL10, an immature form of rhIL-10 that contains the glycosylation-modified signal peptide, remained in the ER for a prolonged period at 30°C. The retention resulted in higher ER stress levels that were accompanied by increased ROS production, Ca^2+^ leakage, ER-containing autophagosomes, shortened cortical ER length and compromised induction of the unfolded protein response (UPR). In contrast, G3-pro-rhIL10 was quickly processed and eliminated from the ER at 20°C, resulting in a lower level of ER stress and improved rhIL-10 production.

**Conclusions:**

High-temperature cultivation of an rhIL-10 expression strain leads to prolonged retention of immature G3-pro-rhIL10 in ER, causing higher ER stress levels and thus greater yeast cell death rates and lower production of rhIL-10.

**Electronic supplementary material:**

The online version of this article (doi:10.1186/s12934-014-0163-7) contains supplementary material, which is available to authorized users.

## Background

The methylotrophic yeast *Pichia pastoris* (*P. pastoris*) is a well-established eukaryotic host for the production of heterologous proteins [1,2]; however, the production yield widely varies among particular proteins. For maximal production of a target protein, increasing target protein synthesis by increasing the gene copy number is a commonly employed strategy, but it is only effective up to a certain gene copy number limit [3] above which further increases can reduce production [4,5]. In contrast, decreasing the synthesis rate of the target protein by lowering the cultivation temperature from 30°C to 20°C is also commonly employed, and it is frequently reported to improve production [6–8]. The effectiveness of these discrepant strategies suggests that heterologous protein production is limited by the capacity of intracellular processes in *P. pastoris*. Understanding the potential mechanisms of this bottleneck will provide information that is useful for rationally engineering strategies to optimize yeast strains and the fermentation process.

Newly synthesized secretory proteins that fail to adopt their correct conformations may be retained in the endoplasmic reticulum (ER). The ER houses a specialized folding environment that includes chaperones and foldase complexes as well as high-fidelity quality control mechanisms to ensure correct protein folding [9,10]. ER homeostasis is defined as the unique equilibrium between the cellular demand for protein synthesis and the folding capacity of the ER, which promotes protein transport and maturation [11]. The retention of immature (also called misfolded) proteins in the ER can induce ER stress and disrupt ER homeostasis. The unfolded protein response (UPR) pathway is activated to mitigate ER stress; the UPR transcriptionally upregulates a group of genes required to increase the ER folding capacity by providing more chaperones and foldases and by increasing the ER surface area and luminal space [12,13]. Thus, the UPR pathway is thought to be adaptive, re-establishing ER homeostasis and normalizing ER function in a changing environment [14]. However, unlike metazoans, the UPR pathway of budding yeast does not include the translational repression sensor PERK, which temporarily inhibits general translation by phosphorylating eIF2α and thus leading to reduced protein entry into the ER lumen [13,15]. Accordingly, the absence of PERK in budding yeast makes it more sensitive to the overexpression of heterologous proteins.

The ER-associated protein degradation pathway (ERAD) and autophagy are two important degradation mechanisms for eliminating potentially toxic misfolded proteins. The ERAD pathway clears folding-defective proteins by facilitating their movement into the cytosol and their subsequent proteolytic degradation by the 26S proteasome [9,16]. ERAD thereby prevents the accumulation of toxic proteins and reactive oxygen species (ROS), reducing cell death [17]. Notably, the ERAD pathway can be saturated by high substrate concentrations, as during CPY* overexpression [18,19]. The alternative degradation pathway, autophagy, engulfs and delivers misfolded proteins to the vacuole for degradation [20–22]. In contrast to non-specific autophagy, ER-phagy, which selectively sequesters protein aggregates containing ER fragments and returns the ER to its normal size once folding stress subsides, is suggested to provide a survival advantage in response to ER stress [20,23,24]. Although autophagy is generally considered to be a mechanism for cell survival, recent studies also indicate its involvement in cell death, as a number of genes required for autophagy are also involved in apoptosis [25,26].

To more deeply understand the effect of cultivation temperature on protein production, a *P. pastoris* strain expressing recombinant human interleukin-10 (rhIL-10) under the control of the *AOX1* promoter was constructed and employed as a model. The cellular responses to ER stresses, including UPR, ERAD and autophagy, were analyzed. The results indicate that ER folding capacity and yeast cell viability are preserved at 20°C, leading to high production of rhIL-10. In contrast, ER stress was induced by prolonged retention of immature G3-pro-rhIL10 at 30°C, leading to impaired ER folding capacity and decreased yeast cell viability and hence to low production of rhIL-10. This study highlights the importance of balancing the synthesis rate of rhIL-10 with the ER folding capacity.

## Results

### High-temperature cultivation of an rhIL-10 expression strain increases cell death

**Figure 1:**
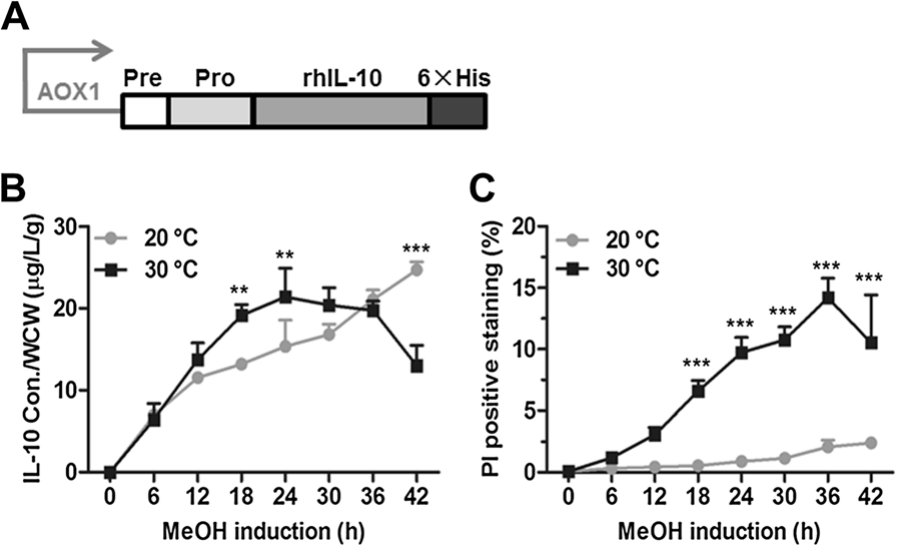
**High-temperature cultivation of an rhIL-10 expression strain increases cell death. (A)** Schematic representation of the rhIL-10 expression cascade. **(B)** The rhIL-10 expression strain (clone ‘H’) was methanol-induced at either 20°C or 30°C in a 10-L fermentor. The production of rhIL-10 was measured by an ELISA assay. **(C)** Yeast cell viability was examined by PI staining and analyzed by flow cytometry. The statistical results are presented as the mean ± SD. (**P <0.01, ***P <0.001).

To generate an rhIL-10 expression strain, an rhIL-10 expression cascade under the control of the *AOX1* promoter was constructed and introduced into the yeast *P. pastoris* (Figure 1A). The selected high-expression recombinant strain was methanol-induced for protein production in parallel 10-L fed-batch fermentations at either 20°C or 30°C. During the methanol induction phase, the cell growth curves at 20°C and 30°C were not significantly different (Additional file 1: Figure S1). Specific production of rhIL-10 (normalized to wet cell weight) and yeast cell viability were monitored by ELISA and PI staining, respectively (Figure 1B and 1C). The results showed clearly distinct patterns for both protein production and cell death rate at 20°C and 30°C. At 20°C, specific production of rhIL-10 continuously increased (Figure 1B), and the cell death rate stayed below 4% throughout the process (Figure 1C). However, at 30°C, production of rhIL-10 displayed a curve with a peak at the 24-h time point that was followed by a continuous decrease (Figure 1B); the cell death rate continuously increased from the initial methanol induction to the end of the experiment (Figure 1C). These significant differences in the cell death rate and the production of rhIL-10 at different culture temperatures prompted us to explore the underlying mechanisms.

### High-temperature cultivation of an rhIL-10 expression strain impairs the maturation of G3-pro-rhIL10

**Figure 2:**
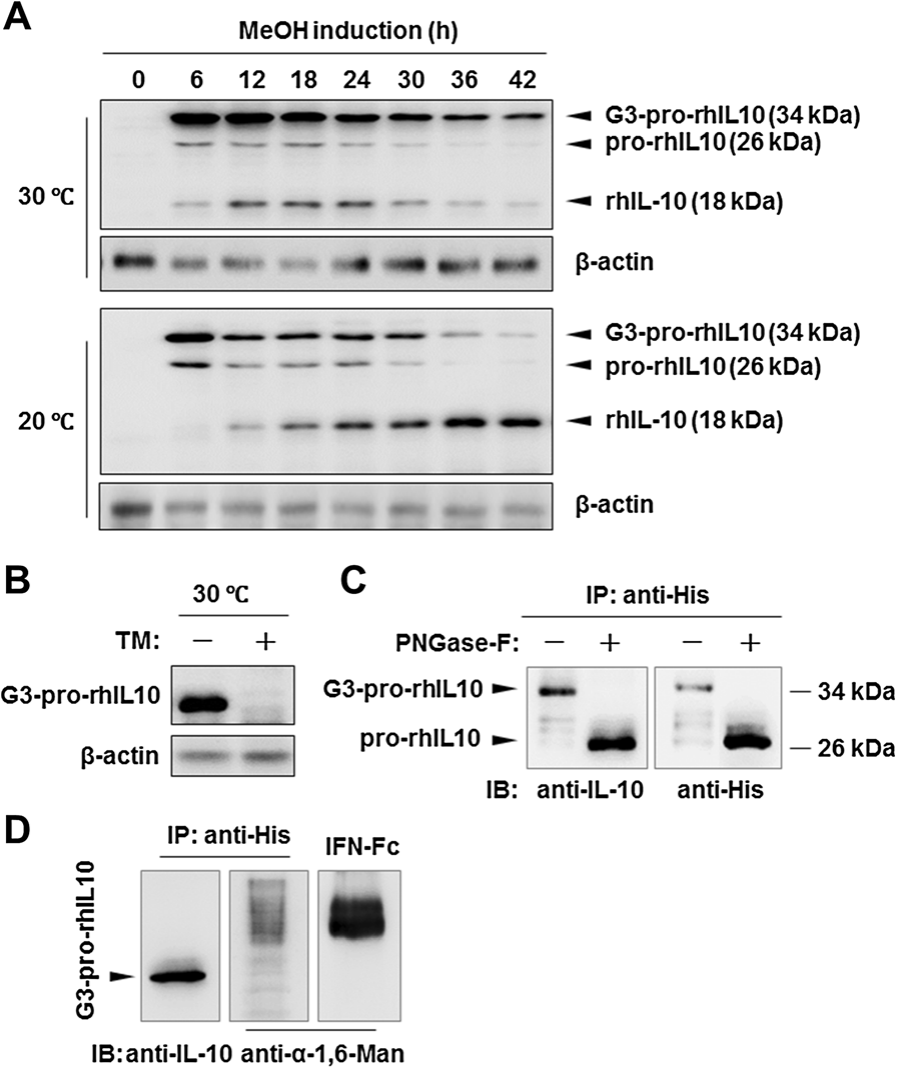
**High-temperature cultivation of an rhIL-10 expression strain impairs the maturation of G3-pro-rhIL10. (A)** Whole-cell lysates were prepared from cells as in Figure 1B and examined by Western blotting using an anti-His antibody. β-actin was used as a loading control. The bands labeled G3-pro-rhIL10, pro-rhIL10 and rhIL-10 represent the intracellular forms of rhIL-10. **(B)** The rhIL-10 expression strain (clone ‘H’) was methanol-induced at 30°C for 24 hours with or without the addition of 5 μM tunicamycin (TM) in shaking flask culture. Whole-cell lysates were examined by Western blotting. **(C)** Intracellular G3-pro-rhIL10 (34 kDa) was initially purified with Ni-affinity beads, de-glycosylated with PNGase F, and finally examined by Western blotting using anti-IL-10 and anti-His antibodies. **(D)** Purified G3-pro-rhIL10 was directly examined by Western blotting with anti-IL-10 and anti-α-1,6-mannose antibodies, and IFN-Fc produced from *Pichia pastoris* (made in our lab) was employed as a positive control. All the experiments were repeated three times and showed similar results.

To explore the mechanisms of increased cell death by high-temperature cultivation, whole-cell lysates from the fermentation samples as in Figure 1B were extracted and examined by Western blotting with a specific anti-His tag antibody. As shown in Figure 2A, three forms of intracellularly retained rhIL-10 (observed by their different molecular weights) were detected. By considering the possible products that could be produced by the coding sequence of the expression construct, these three different product forms were accordingly designated as two immature forms, G3-pro-rhIL10 (34 kDa) and pro-rhIL10 (26 kDa), which contain an α-factor pro-peptide with or without three sites of N-glycosylation (further characterized in Figure 2B and 2C), respectively, and as the mature form of rhIL-10 (18 kDa, without the α-factor pro-peptide). Interestingly, at 20°C, the levels of G3-pro-rhIL10 and pro-rhIL10 gradually decreased with a concurrent increase of the mature form of rhIL-10 (18 kDa); however, at 30°C, G3-pro-rhIL10 was consistently present throughout the course of methanol induction and was accompanied with a rapid decrease in the mature form of rhIL-10 after the 24 h time-point (Figure 2A). Taken together with the data in Figure 1, the failed maturation of G3-pro-rhIL10 at 30°C suggested that significant impairment of the protein processing machinery might be occurring.

To characterize the intracellularly retained forms of rhIL-10, the rhIL-10 expression strain was treated at 30°C with or without the N-glycosylation inhibitor tunicamycin (TM) [27]. TM treatment inhibited the formation of G3-pro-rhIL10 (Figure 2B). In addition, as shown in Figure 2C, G3-pro-rhIL10, when purified using Ni-affinity beads from the whole cell lysates, was digested by the PNGase-F enzyme from the 34–kDa form (G3-pro-rhIL10) into the 26-kDa form (pro-rhIL10). Thus, the results clearly demonstrate that G3-pro-rhIL10 is an immature form of the protein that retains the α-factor pro-peptide containing three consensus N-glycosylation sites, as previously reported [28].

The processing of α-factor pre-pro-peptide involves the removal of the pre-peptide by signal peptidase in the endoplasmic reticulum and the cleavage of the pro-peptide by the Kex2 endopeptidase in the late Golgi [29,30]. When misfolded proteins are retained in the early Golgi, α-1,6-mannose will be linked to the glycans of proteins [10]. To characterize the specific subcellular location of G3-pro-rhIL10, purified G3-pro-rhIL10, together with IFN-Fc protein as a positive control, were examined by Western blotting using a specific antibody against α-1, 6-mannose. The experiment detected no specific signals for α-1,6-mannose on G3-pro-rhIL10 (Figure 2D), indicating that the G3-pro-rhIL10 was not located in the early Golgi but was most likely in the ER.

### High-temperature cultivation of an rhIL-10 expression strain increases ER stress

**Figure 3:**
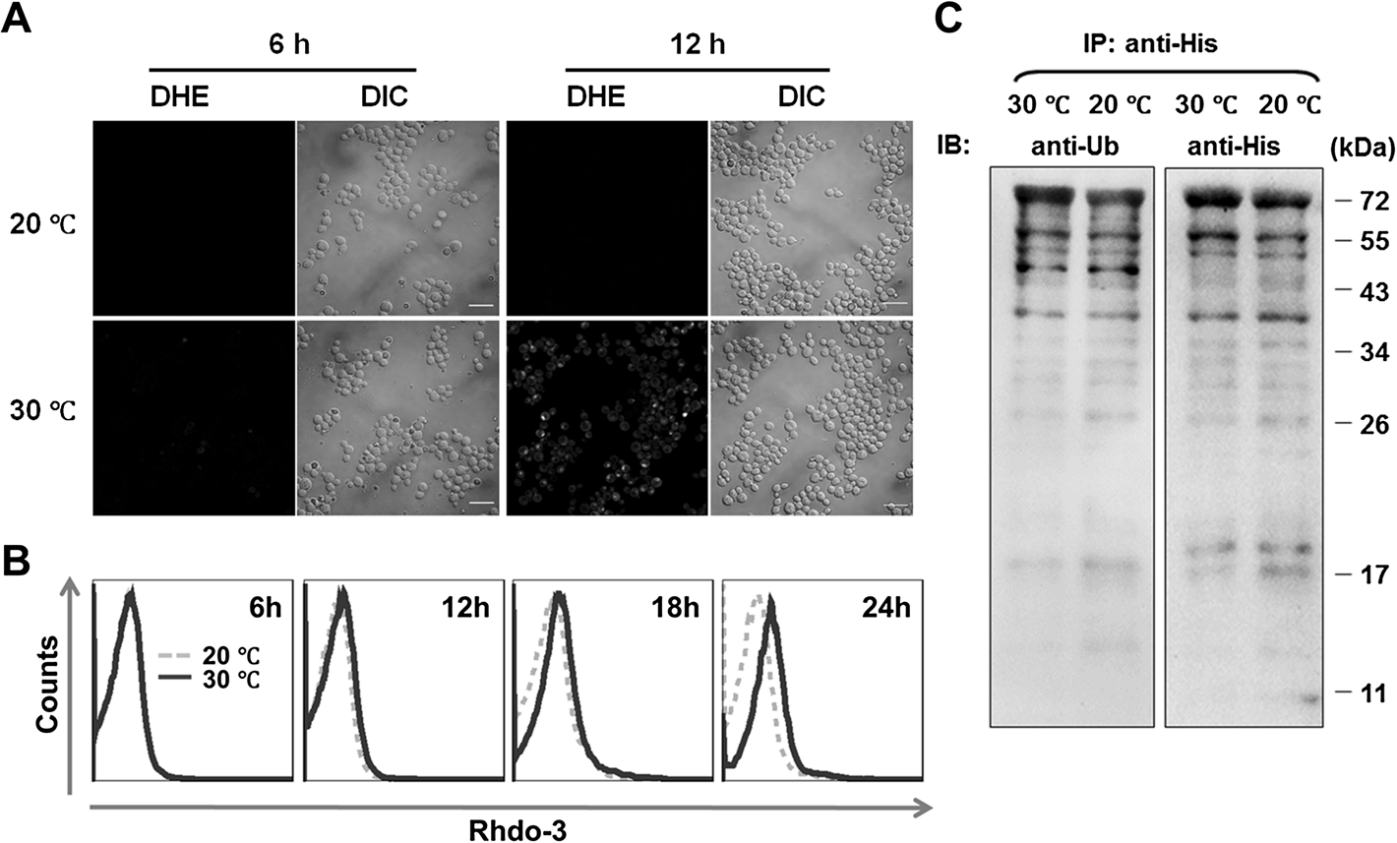
**High-temperature cultivation of an rhIL-10 expression strain increases ER stress. (A)** Reactive oxygen species (ROS) were monitored with dihydroethidium (DHE) and visualized by confocal fluorescence microscopy. **(B)** Cytosolic Ca^2+^ levels were monitored by staining with Rhod-3/AM and examined by flow cytometry. **(C)** Ubiquitin-conjugated rhIL-10 was purified from whole-cell lysates with Ni-affinity beads (cells normalized to an equivalent of 0.1 OD600 units) and examined by Western blotting using anti-Ub and an anti-His antibodies. All experiments were repeated three times and showed similar results. Scale bars: 10 μm.

The prolonged retention of misfolded proteins in the ER can induce ER stress and cause the accumulation of reactive oxygen species (ROS) [31]. To compare the accumulated ROS levels at 20°C and 30°C, yeast cells were stained with dihydroethidium (DHE) and examined by confocal microscopy. The yeast cells grown at 30°C clearly showed greater DHE staining than the cells grown at 20°C at the 12 h time point (Figure 3A), indicating that ROS levels were higher at 30°C than at 20°C. In addition, ER stress can lead to the release of Ca^2+^ from the ER into the cytoplasm. Thus, to further compare the level of ER stress at 20°C and 30°C, cytosolic Ca^2+^ was stained with Rhod-3/AM and examined by flow cytometry. The result showed that the level of cytosolic Ca^2+^ at 30°C gradually increased, and a difference between cytosolic Ca^2+^ levels at 20°C and 30°C gradually became evident, especially at the 24-h time point (Figure 3B). Taken together with the data in Figure 2A, these results strongly suggested that the rhIL-10 expression strain experienced greater ER stress at 30°C than at 20°C.

ERAD provides a clearance mechanism to remove ER-retained misfolded proteins, thus aiding in maintaining the folding capacity and secretory efficiency of the ER [16]. To examine and compare the status of the ERAD machinery at 20°C and 30°C, ubiquitin-conjugated rhIL-10 forms were isolated using Ni-affinity beads and examined by Western blotting using an anti-ubiquitin antibody. As shown in Figure 3C, the levels of ubiquitin-conjugated rhIL-10 forms at 20°C were equivalent to those at 30°C, indicating that the ERAD machinery in the rhIL-10 expression strain was saturated at both 20°C and 30°C.

### High-temperature cultivation of an rhIL-10 expression strain impairs ER folding capacity

**Figure 4:**
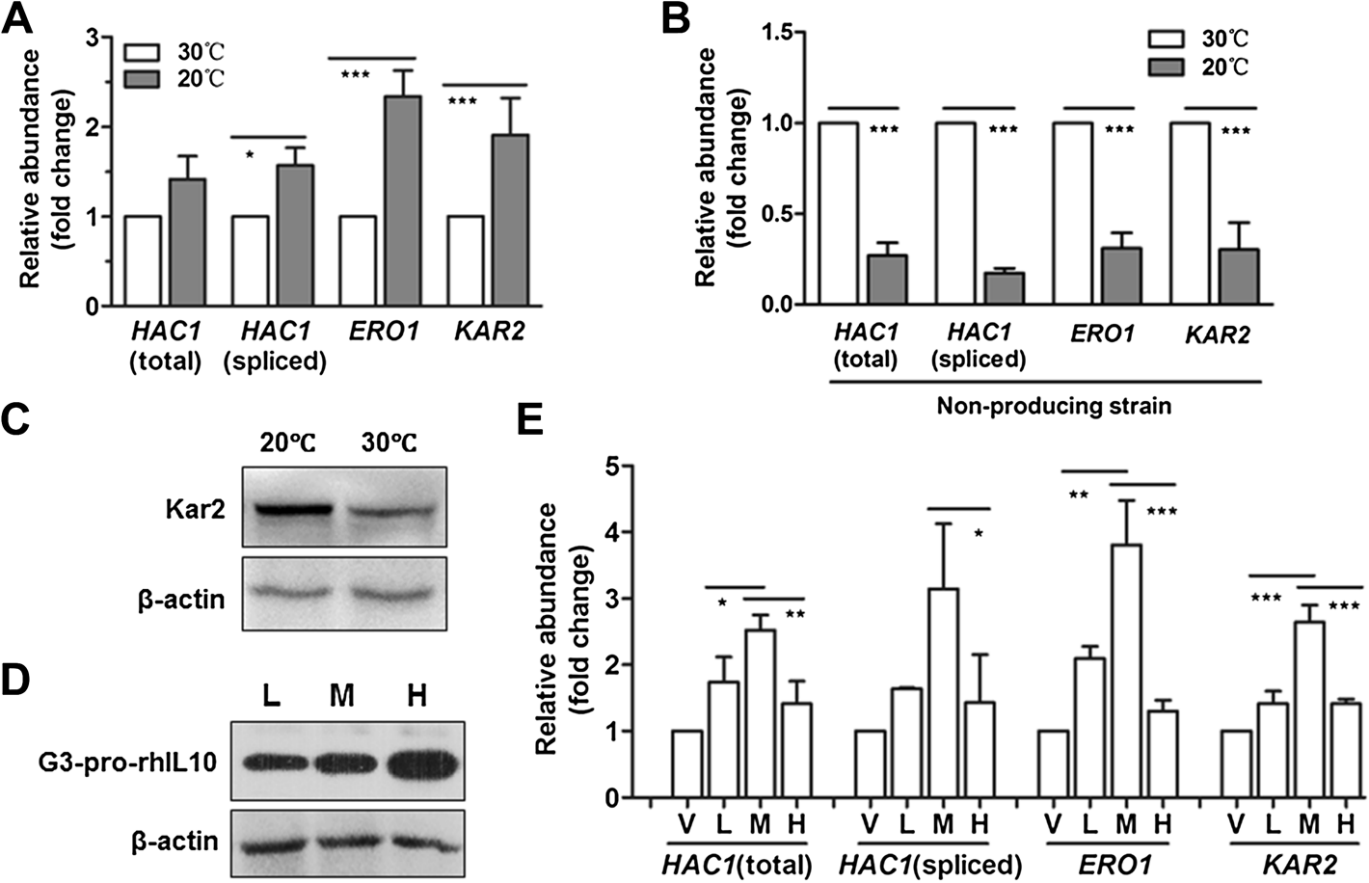
**High-temperature cultivation of an rhIL-10 expression strain impairs ER folding capacity. (A)** Total RNA was extracted from yeast cells at the 24-h time point as in Figure 1B, and the mRNA levels of primary UPR genes were examined by qRT-PCR. **(B)** Changes in UPR induction in a non-producing strain. The non-producing strain was methanol-induced for 24 hours before extraction of total RNA. The mRNA levels of primary UPR genes were examined by qRT-PCR. **(C)** Whole-cell lysates from the 24-h time point as in Figure 2A were examined by Western blotting using an anti-Kar2 antibody, with β-actin as a loading control. **(D)** The rhIL-10 expression strains were methanol-induced for 24 hours at 30°C in shaking flask culture. Whole-cell lysates (extracted from a volume of cells equivalent to 0.1 OD600 units) were examined by Western blotting. The labels ‘L,’ ‘M’ and ‘H’ indicate 1, 5 and 10 gene copies, respectively. **(E)** Total RNA was extracted from the cells as in Figure 4D, and the mRNA levels of primary UPR genes were examined by qRT-PCR. The label ‘V’ indicates the vector control. All the experiments were repeated three times and showed similar results. The statistical results presented represent the mean ± SD. (*P <0.05, **P <0.01, ***P <0.001).

As the level of G3-pro-rhIL10 was higher at 30°C than at 20°C (Figure 2A), we assumed that the UPR might be more strongly induced at 30°C than at 20°C to enable cell survival. To examine the status of the UPR, total RNA was extracted from the samples at the 24-h time point (as in Figure 2A) and subjected to qRT-PCR. Unexpectedly, the relative mRNA levels of primary UPR genes, including *HAC1* (spliced forms), *ERO*1 and *KAR2*, were significantly lower in the rhIL-10 expression strain at 30°C than at 20°C (Figure 4A). However, the relative mRNA levels of primary UPR genes in a non-producing strain were significantly higher at 30°C than at 20°C (Figure 4B), in accord with previous reports [7,8]. Consistent with the changes in mRNA levels shown in Figure 4A, the relative protein level of Kar2p in the rhIL-10 expression strain was lower at 30°C than at 20°C (Figure 4C). As molecular chaperones and foldases are critically important for correct protein folding, these data indicated that the ER folding capacity of the rhIL-10 expression strain was higher at 20°C than at 30°C, suggesting that UPR induction in the rhIL-10 expression strain might be impaired at 30°C.

To further confirm the data shown in Figure 4A, rhIL-10 expression strains with different copy numbers of the rhIL-10 expression cascade (typically 1, 5, and 10 copies, see the Methods section) were cultured in shaking baffled flasks at the same cultivation temperature. As shown in Figure 4D, the levels of ER-retained G3-pro-rhIL10 were positively correlated with rhIL-10 copy number. Importantly, the relative mRNA levels of the primary UPR genes from the strain with 10 copies (labeled ‘H’) were significantly lower than in a strain with 5 copies (labeled ‘M’), as shown in Figure 4E. These results suggested that UPR induction was impaired once the level of ER-retained G3-pro-rhIL10 exceeded a certain threshold.

### High-temperature cultivation of an rhIL-10 expression strain induces ER-phagy

**Figure 5:**
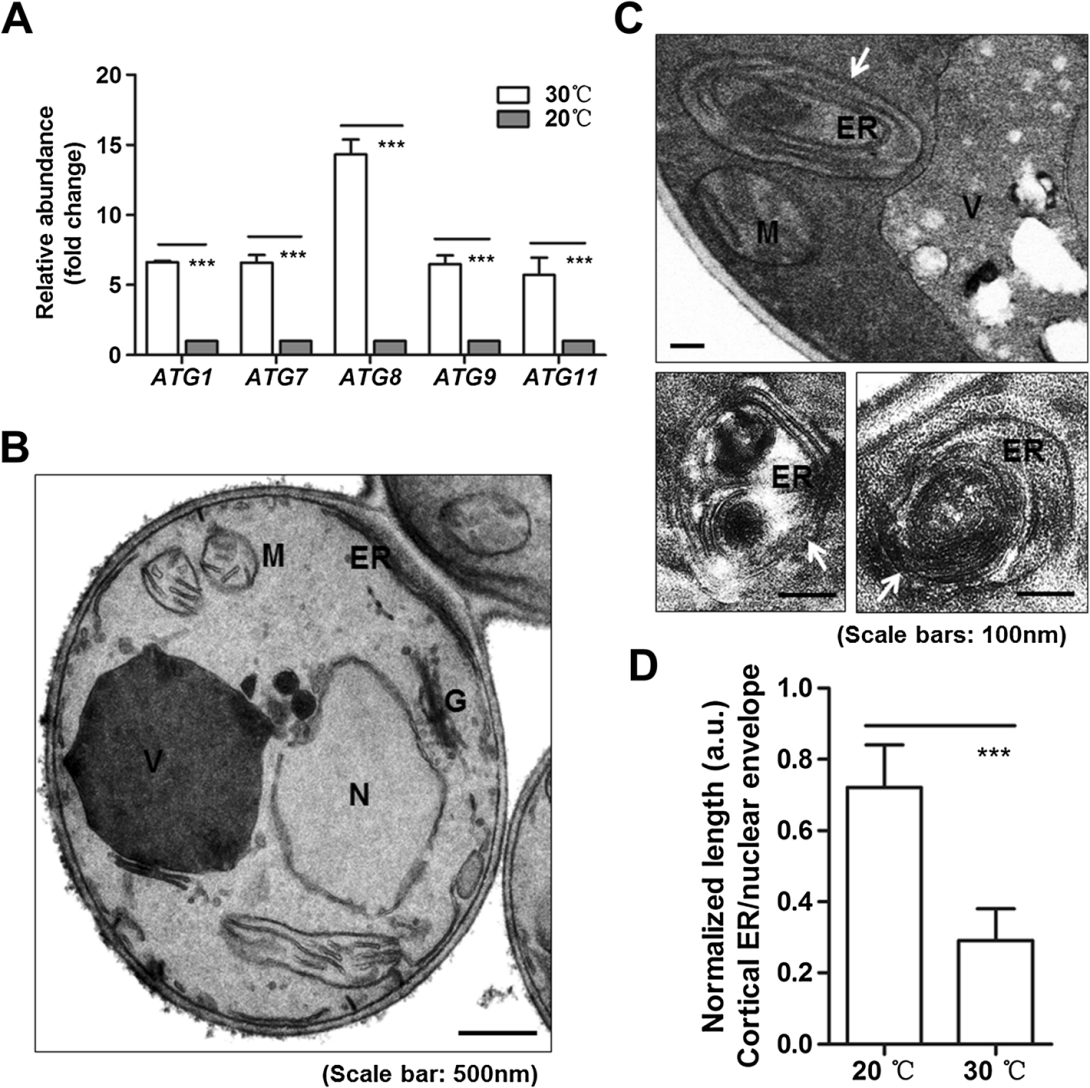
**High-temperature cultivation of an rhIL-10 expression strain induces ER-phagy. (A)** The rhIL-10 expression strain (clone ‘H’) was methanol-induced for 24 hours. Total RNA was extracted, and the mRNA levels of primary autophagy genes were examined by qRT-PCR. The statistical results are based on three independent experiments and represent the mean ± SD (***P <0.001). **(B)** A typical transmission electron micrograph of a yeast cell at 20°C. **(C)** Typical transmission electron micrographs of yeast cells at 30°C. ER-containing autophagosomes (ERAs) are labeled with white arrows. Vacuoles, nuclei, endoplasmic reticula, Golgi and mitochondria are labeled as ‘V,’ ‘N,’ ‘ER,’ ‘G’ and ‘M,’ respectively. **(D)** The normalized length of the cortical ER was measured using transmission electron micrographs. Data represent the mean ± SD (***P <0.001).

Autophagy is a crucial degradation pathway that is involved in preserving ER functionality [20], and thus autophagy is largely viewed as a pro-survival mechanism in response to ER stress. To compare the autophagic activity in the rhIL-10 expression strain at 20°C and 30°C, total RNA was extracted and subjected to qRT-PCR. The results showed that the relative mRNA levels of autophagy pathway genes, including *ATG1*, *ATG7*, *ATG8*, *ATG9* and *ATG11*, were significantly higher at 30°C than at 20°C (Figure 5A). These data suggested that autophagic activity was induced to a higher level at 30°C, in accord with the higher level of ER-retained G3-pro-rhIL10 at 30°C.

To further confirm autophagic activity, yeast cells were subjected to ultra-thin sectioning and examination by transmission electron microscopy (see the Methods section). A typical cell image from 20°C growth is presented in Figure 5B; no detectable autophagic structure was observed. Unexpectedly, ER-phagic structures (ER-containing autophagosomes) were observed at 30°C (labeled by white arrow, Figure 5C). Double-membraned autophagosomes in particular were filled with stacked membrane cisternae (Figure 5C), consistent with the criteria for the morphological definition of ER-phagy [20,24]. Moreover, the statistical data in Figure 5D showed that the normalized length of the cortical ER was significantly shorter at 30°C than at 20°C, consistent with the data shown in Figure 5B and 5C. Collectively, these results suggest that high-temperature cultivation of the rhIL-10 expression strain at 30°C causes severe ER stress that might induce ER damage and thus ER-phagy.

## Discussion

Improved protein production during low-temperature cultivation is generally attributed to higher yeast cell viability [6,32], lower proteolytic activity against the target protein and decreased folding stress [7,8]. In contrast, the mechanisms of decreased production during high-temperature cultivation are unfortunately often neglected. In this study, we provide evidence to demonstrate that high-temperature cultivation of an rhIL-10 expression strain leads to prolonged accumulation of immature G3-pro-rhIL10 in ER, thereby inducing higher levels of ER stress and subsequent cell death. However, lowering the production rate of rhIL-10 by low-temperature cultivation effectively mitigates ER stress, preserving the folding capacity of the ER and enhancing cell viability, in accord with other reports about the positive effects of low-temperature cultivation.

It might be expected that the induction of the UPR corresponds with the misfolded protein level, but such a correspondence was not observed in this study. A series of cellular processes, including accumulation of immature G3-pro-rhIL10 (Figure 2A), shortened cortical ER length (Figure 5D), ER-containing autophagosomes (Figure 5C) and compromised UPR induction (Figure 4A) were found when the rhIL-10 expression strain was cultured at 30°C but not when it was grown at 20°C. Considering that the UPR pathway sensor Ire1 and multiple chaperones (such as Kar2) reside in the ER membrane and lumen, respectively, sequestering and degrading the cortical ER by ER-phagy can reduce signaling in the UPR pathway. Thus, it would not be surprising to see relatively lower UPR induction at 30°C, the temperature at which the immature G3-pro-rhIL10 protein accumulated for a prolonged period and was accompanied in this study by activated ER-phagy.

Due to the specific properties of each target protein, the folding and processing efficiency of each target protein in the ER will be different. Human interleukin-10 contains two intrachain disulfide bridges, and its natural conformation is a non-covalent homodimer [33], which is subject to misfolding when overexpressed. Thus, the severe physiological effects caused by ER-retained immature G3-pro-rhIL10 in this study might be specific for the expression of rhIL-10 and not for heterologous protein production in general.

## Conclusions

**Figure 6:**
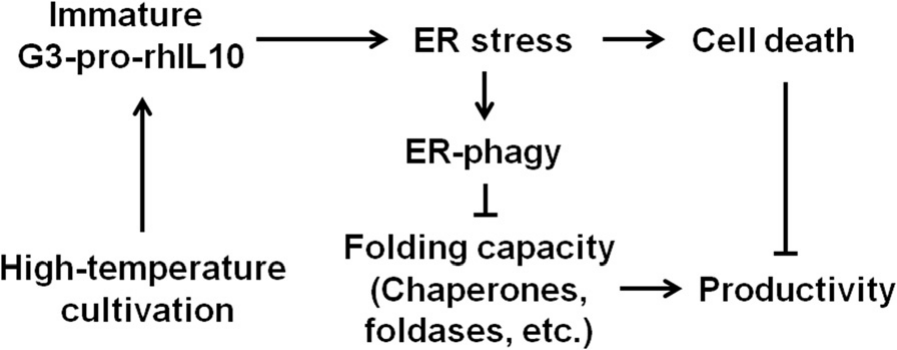
**Model of decreased rhIL-10 production due to high-temperature cultivation.** High-temperature cultivation of an rhIL-10 expression strain leads to the prolonged accumulation of immature G3-pro-rhIL10 in ER, thus increasing the level of ER stress. Increased ER stress then disrupts the ER folding capacity by activating ER-phagy and increases yeast cell death.

In this study, we provide detailed data on the cellular physiology of recombinant *P. pastoris* to explore the low production of rhIL-10 by high-temperature cultivation. Most importantly, we provide solid evidence to show that high-temperature cultivation of an rhIL-10 expression strain leads to the prolonged accumulation of immature G3-pro-rhIL10 in the ER. This accumulation results in a higher level of ER stress, which disrupts ER folding capacity by activating ER-phagy and increases yeast cell death (Figure 6). This study highlights the importance of compatibility between the production rate of rhIL-10 and the ER folding capacity of recombinant *P. pastoris*, a relationship that would also hold true for other heterologous proteins.

## Methods

### Reagents

SYBR-Premix Ex Taq (DRR420A) was purchased from Takara (Dalian, China). Yeast extract (LP0021) and peptone (LP0037) were purchased from Oxoid (UK). pPIC9K (V17520), GS115 (K174001), propidium iodide (P3566), the Rhod-3 Calcium Imaging Kit (R10145), Pluronic F-127 (P-3000MP) and SuperScript III (18080051) were purchased from Invitrogen (USA). Tunicamycin (TF1129) was purchased from Sangon Biotech (Shanghai, China). Human IL-10 ELISA Kits (D1000B) and the antibody for rabbit IgG (HAF008) were purchased from R&D Systems (USA). DNase I (EN0525), *Bam*HI (ER0055), *Not*I (ER0591) and Pierce ECL Western Blotting Substrate (32106) were purchased from Thermo Scientific (USA). The His-tag antibody (M20001) was purchased from Abmart (Shanghai, China). The mouse IgG antibody (7076) was purchased from Cell Signaling (USA). The β-actin (bs-0061R) antibody was purchased from Bioss (Beijing, China). The ubiquitin antibody (ab24686) was purchased from Abcam (USA). The human IL-10 antibody (sc-8438) was purchased from Santa Cruz Biotechnology (USA). The α-1,6-Man antibody (RSY919) was kindly provided by Howard Riezman (University of Geneva). The Kar2 antibody was kindly provided by Randy Schekman (UC Berkeley). The glutaraldehyde (02607-BA), uranyl acetate (02624-AB), lead citrate (02616-AA) and Spurr kits (02680-AB) were purchased from SPI (USA). Antifoam-204 (A8311), yeast nitrogen base (Y1251), RNase A (R6513), Ni-affinity beads (P6611), dihydroethidium (D7008), G418 (A1720) and glass beads (G8772) were purchased from Sigma (USA). All other chemical reagents were purchased from Sinopharm Chemical Reagent Co., Ltd (Shanghai, China).

### Plasmid construction

For the expression of recombinant human IL-10 (rhIL-10), plasmid pZYJ1 was constructed by inserting an expression cascade containing the human IL-10 coding region (NP_000563.1, 19-178 aa) and encoding an α-factor pre-pro-peptide at the N-terminus and a 6XHis-tag at the C-terminus into the *Bam*HI and *Not*I restriction enzyme sites of pPIC9K.

### Recombinant strains

To generate the rhIL-10 expression strains, the histidine-auxotrophic *P. pastoris* strain GS115 was used in this study. The rhIL-10 expression strains with different copy numbers of the rhIL-10 expression cascade were generated by transforming pZYJ1 into strain GS115 and screening the transformants on G418 plates with gradually increasing G418 concentrations from 0.2 mg/mL to 1 mg/mL, as previously described [34].

### Cell culture

For shaking flask cultures, a single colony was inoculated into 5 mL BMGY (1% yeast extract, 2% peptone, 1.34% YNB without amino acids, 1% glycerol, 0.4 mg/L biotin and 0.1 M potassium phosphate, pH 6.0) and grown overnight at 30°C and 200 rpm in a shaking incubator. Aliquots of these cultures, corresponding to a final OD600 of 0.5, were transferred to 10 mL BMGY and incubated at 30°C and 200 rpm. After reaching exponential growth phase, the cells were harvested by centrifugation at 1,500 x *g* for 5 min at room temperature. The pellet was resuspended in BMMY medium (1% yeast extract, 2% peptone, 1.34% YNB without amino acids, 0.4 mg/L biotin, 1% methanol and 0.1 M potassium phosphate, pH 6.0) and was incubated for the indicated time and at the indicated temperature at 250 rpm.

For fed-batch fermentation, a 1-mL cryostock of recombinant yeast cells was inoculated into 200 mL BMGY in a 2-L shake flask. The cultures were grown overnight at 30°C with shaking at 200 rpm and were transferred into a 14-L Benchtop Fermentor (NBS, BioFlo 115) containing 6 L BMGY medium to a final OD600 of 1.0. After exhaustion of the batch-phase glycerol, indicated by a dissolved oxygen (DO) spike, the glycerol fed-batch phase and the methanol fed-batch phase were initiated following the *Pichia* fermentation guidelines (Invitrogen). The cultivation temperature for methanol-induced expression was maintained at either 20°C or 30°C, and the pH was maintained at 6.0 with 25% ammonium hydroxide. The DO concentration was maintained at approximately 30% saturation by automatic adjustment of the stirrer speed between 600 and 1000 rpm and by mixing pure oxygen with air if required. Antifoam-204 was added to suppress foaming. Wet cell weights (WCW) were determined every 6 h from the beginning of the fed-batch phase.

### Flow cytometry

The yeast cell viability assay was performed as previously described by propidium iodide (PI) staining [35]. The samples were mixed for 10 seconds immediately prior to the flow cytometry assay. For determination of cytosolic Ca^2+^ levels, yeast cells were incubated in PBS (pH 7.4) with 50 μM Rhod-3/AM at 37°C for 30 min. A non-cytotoxic detergent, Pluronic F-127 (0.5%), was added to increase the solubility of the Rhod-3/AM.

### RNA extraction and cDNA synthesis

Before RNA isolation, all the yeast strains were methanol-induced for 24 hours at the indicated temperatures. The metabolic activity of each culture was immediately quenched after sampling [36]. Total RNA was isolated as previously described [37]. In brief, the cell pellet was resuspended in sodium acetate buffer (50 mM sodium acetate pH 5.2 and 10 mM EDTA pH 8.0) in the presence of 10% SDS and acid-washed glass beads and was then vortex-lysed with hot acidic phenol (pH 4.5-5.5, 65°C). Centrifugation was performed after the addition of chloroform:isoamyl alcohol (24:1), and then the upper aqueous layer underwent ethanol precipitation to obtain the precipitated RNA. To eliminate remaining genomic DNA, the RNA samples were treated with DNase I. cDNA was synthesized using Superscript III according to the manufacturer’s instructions.

### qRT-PCR

Quantitative real-time PCR was performed using SYBR-Premix Ex Taq in a PikoReal 96 Real-Time PCR System (Thermo Scientific, USA) according to the manufacturer’s instructions. For examination of the relative changes in mRNA levels, the *ACT1* gene was used as a reference. The detected target genes and their corresponding primers are shown in Additional file 2: Table S1. All reactions were performed in triplicate. The data were normalized using the average of the internal standard, as previously described [36].

### Isolation of yeast genomic DNA

Yeast genomic DNA was prepared as previously described [38]. In brief, the cell pellet was resuspended in STES buffer (0.2 M Tris, pH 7.6, 0.5 M NaCl, 0.01 M EDTA, 0.1% SDS) and vortex-lysed with phenol:chloroform:isoamyl alcohol (25:24:1) and acid-washed glass beads. After centrifugation, the upper aqueous layer was used for ethanol precipitation. The DNA pellet was further subjected to the elimination of RNA using RNase A and was then purified by phenol:chloroform (1:1) extraction followed by ethanol precipitation. The DNA concentration was measured at 260 nm, and the DNA quality was checked by gel electrophoresis. Only DNA samples displaying 260 nm/280 nm ratios greater than 1.8 were used for further analysis.

### Copy-number assay

The copy numbers of rhIL-10 strains were measured by qPCR as described previously [39]. In brief, genomic DNA was isolated (as described above) and subjected to qPCR with primer pairs specific for the AOX1 promoter and the endogenous ARG4 gene, which served as an internal reference (see Additional file 2: Table S1 Primers). The yeast strain X-33, with one copy of the AOX1 promoter, was employed as the quantity reference group. Recombinant strains with 1, 5 and 10 copies of the cascade encoding rhIL-10 were labeled as ‘L,’ ‘M’ and ‘H,’ respectively (see Additional file 3: Table S2).

### ELISA

The cultures from fed-batch fermentation were centrifuged at 12,000 × *g* for 10 min at 4°C, and the rhIL-10 in the supernatant was measured with ELISA kits by following the manufacturer’s instructions (R&D Systems, D1000B).

### Western blotting

Whole-cell lysates were prepared using an alkaline-lysis method as previously described [40]. Whole-cell lysates were first separated by SDS-PAGE and then transferred to PVDF membranes for Western blotting analysis. Immunoreactive bands were visualized with the Pierce ECL Western Blotting Substrate, and the signals were detected and analyzed with the Alliance 4.7 program (UVItec).

### Fluorescence microscopy

Fluorescent images were captured using a confocal fluorescence microscope. The images were processed using Adobe Photoshop CS3. Image brightness was enhanced by adjusting the levels and curves in Photoshop. For ROS determination, intracellular superoxide anions were measured using DHE as previously described [41].

### Transmission electron microscopy

Yeast cells were prepared as previously described [42]. Briefly, the cells were chemically fixed with glutaraldehyde and potassium permanganate, stained with uranyl acetate, infiltrated with Spurr’s resin, subjected to ultra-thin sectioning and stained with Reynold’s lead citrate. The appropriately treated and stained sections were visualized on an electron microscope (FEI Tecnai F20). The images were processed and analyzed using ImageJ software (W. S. Rasband: http://imagej.nih.gov/ij/). To quantify cortical ER length, the method of normalizing the length of the cortical ER to the length of the nuclear envelope was employed, as previously described [24]. A minimum of 50 cells was counted for each temperature group.

### Statistical analysis

Student’s *t*-test or one-way ANOVA were used where appropriate. *P <0.05 was considered to indicate a statistically significant difference. Data are reported as the mean values of at least three independent experiments.

## Additional files

Additional file 1: Figure S1. Cell growth curves of an rhIL-10 expression strain during the methanol induction phase. Additional file 2: Table S1. Primers for quantitative real-time PCR (5’-3’). Additional file 3: Table S2. Quantitation of rhIL-10 copy number.

## Abbreviations

ER: Endoplasmic reticulum
UPR: Unfolded protein response
ROS: Reactive oxygen species
ERAD: ER-associated protein degradation pathway
PERK: RNA-dependent protein kinase-like ER kinase
rhIL-10: recombinant human interleukin-10
TM: Tunicamycin
DHE: Dihydroethidium
*P. pastoris*: *Pichia pastoris*
WB: Western blotting
Man: Mannose
Ub: Ubiquitin
MeOH: Methanol
IP: Immunoprecipitation
IB: Immunoblotting
WCW: Wet cell weight
