# Supplementary figures and images for: High-temperature cultivation of recombinant Pichia pastoris increases endoplasmic reticulum stress and decreases production of human interleukin-10
Source: Microb Cell Fact. 2014 Nov 26;13:163. doi: 10.1186/s12934-014-0163-7 (PMC4251845; doi:10.1186/s12934-014-0163-7)

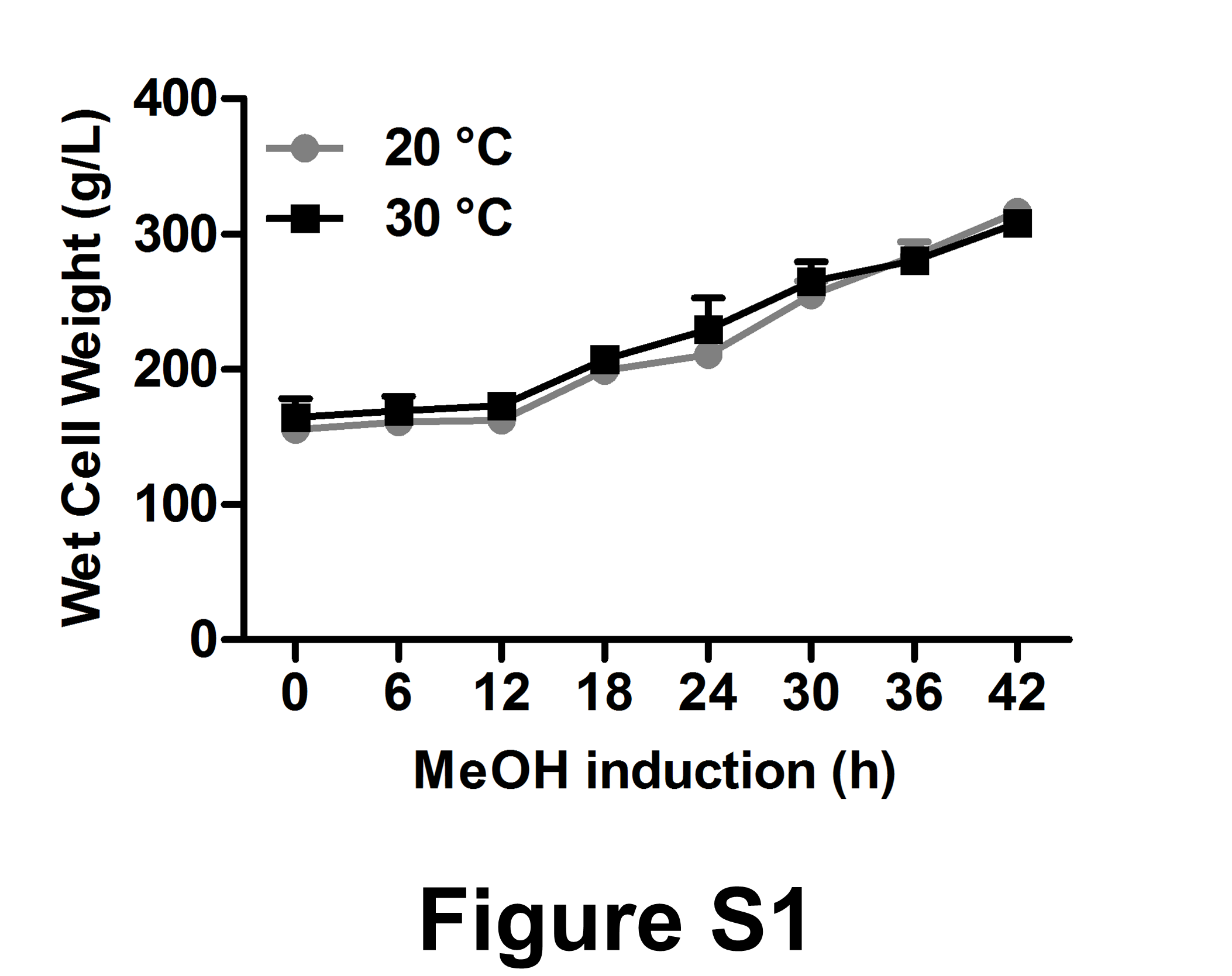

Supplement: Additional file 1: Figure S1. — Cell growth curves of an rhIL-10 expression strain during the methanol induction phase. [file 12934_2014_163_MOESM1_ESM.tiff]
