# Supplementary material for: High-temperature cultivation of recombinant Pichia pastoris increases endoplasmic reticulum stress and decreases production of human interleukin-10
Source: Microb Cell Fact. 2014 Nov 26;13:163. doi: 10.1186/s12934-014-0163-7 (PMC4251845; doi:10.1186/s12934-014-0163-7)
Supplement: Additional file 2: Table S1. — Primers for quantitative real-time PCR (5’-3’). [file 12934_2014_163_MOESM2_ESM.pdf]

Table S1: Primers for quantitative real-time PCR (5’-3’)

HAC1 (spliced)-Fw	AGAGTCCGATGAGAACTTCTTGTTGAGTTC
HAC1 (spliced)-Rv	GCGGTAAATGGTGCTGCTGGATGATG
HAC1 (total)-Fw	GCGGCCCATGCTTCCAGAGAG
HAC1(total)-Rv	CGGTACCACCTAAGGCTTCCAACC
ACT1-Fw	GTGCAGCCTCGCGCTTGTTT
ACT1-Rv	CTGTGTGTGGGGCGTCGTCT
KAR2-Fw	TGGAGAACTACGCTCATTCCCTTAGGA
KAR2-Rv	TGGTTGCGGTGTCGAAGTTGTCT
ERO1-Fw	GTTGGAAAAGCCGCATATAAACAAAACA
ERO1-Rv	CAGCTTGGGCAAAGTCCTGTAAGAGTTC
AOX1-Fw	TACACCACCGCTCTTTTG
AOX1-Rv	TCTCGTAAGTGCCCAACTT
ARG4-Fw	TCCTCCGGTGGCAGTTCTT
ARG4-Rv	TCCATTGACTCCCGTTTTGAG
ATG1-Fw	TCCGGAGAATAGCAAAGGCCTGAA
ATG1-Rv	AACCGGAGGTGAAAGTAGCAGGTT
ATG7-Fw	TGAGCCTCTTACTTCCGAGCCAAA
ATG7-Rv	ACCAAAGCACCTATATCGGCCAGT
ATG8-Fw	AATGCGGATCCGGTACAAGTTTGC
ATG8-Rv	TTAGGTCGCATGGAACCAGGTACT
ATG9-Fw	GTCCACAACCCGTTGTTTGCTGAT
ATG9-Rv	TGGTTTGGAGTCCTCTTCATCGCT
ATG11-Fw	ATCAACCTTGGGCGGCTTTCAATG
ATG11-Rv	TGCTTTCAATCCGTGCAAGAACCC
