# Supplementary material for: High-temperature cultivation of recombinant Pichia pastoris increases endoplasmic reticulum stress and decreases production of human interleukin-10
Source: Microb Cell Fact. 2014 Nov 26;13:163. doi: 10.1186/s12934-014-0163-7 (PMC4251845; doi:10.1186/s12934-014-0163-7)
Supplement: Additional file 3: Table S2. — Quantitation of rhIL-10 copy number. [file 12934_2014_163_MOESM3_ESM.pdf]

Table S2: Quantitation of copy numbers

Sample	C_T (AOX1)	C_T (ARG4)	ΔC_T	$\Delta\Delta C_T$	2^- $\Delta\Delta C_T$	Copy Number
X-33	22.744±0.743	22.781±0.800	-0.037±0.082	0	1	0
L	22.065±0.725	23.100±0.768	-1.035±0.153	-0.999±0.073	2.000±0.100	1.000±0.100
M	20.008±0.736	22.605±0.560	-2.597±0.194	-2.560±0.119	5.912±0.500	4.912±0.500
H	19.448±0.890	22.949±0.725	-3.501±0.170	-3.464±0.123	11.061±0.933	10.061±0.933

Data represents Mean ± SD, n = 3.
